# Supplementary material for: Cochlear Ribbon Synapses in Aged Gerbils
Source: Int J Mol Sci. 2024 Feb 27;25(5):2738. doi: 10.3390/ijms25052738 (PMC10931817; doi:10.3390/ijms25052738)
Supplement: Supplementary file 1 [file ijms-25-02738-s001.zip › ijms-2825946-supplementary.pdf]

Table S1: List of individual specimens and number of IHC evaluated from each.
Columns list, from left to right: Animal ID (publ = part of the dataset in Zhang et al., 2018), age, sex, numbers of IHCs evaluated from each of the three cochlear locations, and total number of IHCs evaluated from a given animal. Colored rows provide summary data for young-adult (blue), quiet-aged (orange), and all gerbils (gray).

gerbil (side)	***Age (days)***	***(months)***	***sex***	2.51 mm 1 kHz	3.8 mm 2 kHz	8.13 mm 16 kHz	**Total IHCs**
Young-adult gerbils:							
AHG 14 (L)	156	5.1	female	11	10	11	**32**
FKSG 24 (R)	228	7.5	female		9	9	**18**
FKSG 25 (R)	134	4.4	male		9		**9**
GLCZ 3 (R) publ	80	2.6	female		9	10	**19**
GLCZ 37 (R) publ	170	5.6	male	5	5	6	**16**
GLCZ 41 (L) publ	205	6.7	male	7	6		
GLCZ 41 (R) publ						4	**17**
GLCZ 43 (L) publ	219	7.2	female	8	7		
GLCZ 43 (R) publ						8	**23**
OG 40 (L)	319	10.5	female	10			**10**
Olivia (R)	273	9.0	female	10			
Olivia (L)						10	**20**
Total young-adult	*9 gerbils / 6 female, 3 male*			*51*	*55*	*58*	*164*
Quiet-aged gerbils							
AddG 19 (L)	1242	40.8	male	8	11	5	**24**
AHG 10 (L)	1163	38.2	female	8			**8**
AHG 24 (R)	1264	41.6	male	8	9		**17**
AHG 25 (R)	1260	41.4	female	9	9	9	**27**
AHG 29 (R)	1170	38.5	male	9			**9**
FKSG 27 (R)	1096	36.0	female			9	**9**
FKSG 29 (L)	1158	38.1	male		10	11	
FKSG 29 (R)				8			**29**
FKSG 30 (R)	1151	37.8	male		9	7	**16**
FKSG 32 (R)	1096	36.0	male		9	8	**17**
Geowaz (L)	1172	38.5	male	8			**8**
Magneton (L)	1212	39.8	male	10		11	**21**
Simsala (R)	1200	39.5	female		9		**9**
Turtok (L)	1269	41.7	male		9	9	**18**
Total quiet-aged	*13 gerbils / 4 female, 9 male*			*68*	*75*	*69*	*212*
Total	***22 gerbils / 10 female, 12 male***			***119***	***130***	***127***	***376***
